# Enrichment of Ginseng Rare Sapogenin 25-OH-PPT and Its Protective Effect on Myocardial Fibrosis

**DOI:** 10.3390/molecules29235813

**Published:** 2024-12-09

**Authors:** Zixuan Jin, Yuemin Wu, Yanyan Zhang, Siqi Feng, Guotao Hu, Hairong Liu, Yuqing Zhao, Jing Xu

**Affiliations:** 1 School of Functional Food & Wine, Shenyang Pharmaceutical University, Shenyang 110016, China; hahahabesun@163.com (Z.J.); wuyuemin1998@163.com (Y.W.); yyx1051800@163.com (Y.Z.); fsq1109@163.com (S.F.); roy02050@163.com (G.H.); clytze0422@126.com (H.L.); 2 Key Laboratory of Natural Medicines of the Changbai Mountain, Ministry of Education, Yanbian University, Yanji 133002, China

**Keywords:** 25-OH-PPT, acid hydrolysis, enrichment, myocardial protection

## Abstract

Ginseng (*Panax ginseng* C. A. Meyer), a traditional Chinese medicine, and the rare ginsenosides contained in it have various physiological activities. 25-OH-PPT (T19) is a rare natural dammarane-type ginseng sapogenin. Pharmacological studies have shown that T19 has good hypoglycemic, antioxidant, and anti-inflammatory activities. In the research, we optimized the T19 enrichment process and explored the potential mechanism of T19 in myocardial oxidative stress. Firstly, we studied a hydrolysis process on ginseng stems and leaves ginsenosides. Optimization factors include acid types, acid concentrations, ultrasound time, and ultrasound temperature. To develop safer preparation conditions more suitable for production scaleup, we studied the difference in hydrolysis between inorganic acid and food acids. The results show that using hydrochloric acid to hydrolyze ginsenosides in ginseng stems and leaves can increase the content of T19 to 12.16%. When using edible citric acid, the maximum content of T19 is 1.9%. However, using citric acid for hydrolysis has higher safety and non-toxic properties. Meanwhile, the myocardial protective effect of T19 was evaluated, indicating that T19 could effectively reduce isoproterenol (ISO)-induced oxidative stress injury by reducing the levels of LDH and CK-MB and regulating the contents of antioxidant enzymes SOD, lipid peroxidation product MDA, and non-enzymatic antioxidant GSH in cardiomyocytes. Further study demonstrated that regulation of fibrosis markers Collagen I, Collagen III, and *α*-SMA was involved in the potential mechanism of T19 efficiency.

## 1. Introduction

Myocardial fibrosis is caused by the accumulation and excessive deposition of fibroblasts and extracellular matrix (ECM) proteins, leading to distortion of organ structure and function [1]. It is an important cause of heart failure and is associated with the renin–angiotensin–aldosterone system, growth factors, inflammatory cytokines, and reactive oxygen species [2,3]. Natural products have been found in pharmacological studies to be effective drugs for the treatment of myocardial fibrosis, including terpenoids, alkaloids, polyphenols, flavonoids, and saponins [4,5,6]. Luo et al. studied the prevention effect of asiaticoside IVa on myocardial fibrosis [7], confirming its attenuation of ISO-induced myocardial fibrosis by activating the expression of target proteins on the AMPK/MTOR/ULK1. Our group found that T19 inhibited H_2_O_2_-induced cardiomyocyte (H9c2) oxidative injury through the PI3K/AKT pathway [8].

Ginseng, the dried root and rhizomes of a perennial plant belonging to the genus *Panax* of the *Araliaceae* family, is commonly used in drugs and food supplements in Asia and America [9,10,11]. Pharmacological studies have shown that ginseng has diverse activities, including antioxidant activities [12], anti-aging [13], regulating blood sugar [14], improving and enhancing the immune system [15], regulating blood pressure [16], and regulating the nervous system [17]. Ginsenosides are the main active constituents of ginseng, which, according to their structure, can be classified as Oleanane-type saponins (OA), Protopanaxadiol-type saponins (PPD), Protopanaxatriol-type saponins (PPT), and Ocotillol-type saponins (OT) [18]. They present good anti-inflammatory, antioxidant, and cardiovascular protective activities [19]. 25-OH-PPT (T19) is a rare ginseng Protopanaxatriol-type sapogenin, isolated and identified by our research group for the first time [20]. Pharmacological studies have shown that T19 has good anti-diabetes, liver inflammation, antioxidant, and blood lipid reduction activities [8,21,22]. Therefore, it is reasonable to expect activity of T19 against myocardial fibrosis.

Modern research has proven that ginsenosides are the main active ingredient in ginseng. However, ginsenosides themselves are not easily absorbed and have low bioavailability. The glycosides, or secondary glycosides, formed by hydrolysis and sugar removal of ginsenosides are more easily absorbed by the body, with enhanced activity and reduced toxicity [23]. T19, as a rare ginseng sapogenin, the content varies in the different parts of ginseng. We established the T19 analysis and detection conditions; the content of T19 in ginseng stems and leaves is the highest [24]. Therefore, we chose total saponins of ginseng stems and leaves as the material for enriching T19. 

The preparation of sapogenin includes physical, chemical, and biological methods. Chemical methods apply acids or alkali for hydrolyzing saponins to remove glycosyl ligands. Previous studies have found that acid hydrolysis has a better enrichment effect on T19 [25]. Therefore, it is important to select a suitable acid and establish a green, non-toxic, and simple method for the enrichment of T19 from ginseng stems and leaves.

This study aims to develop a novel, green, non-toxic, and simple method for enriching T19, and the obtained T19 was subsequently evaluated for the protective effect on myocardial fibrosis.

## 2. Results

### 2.1. Optimization of T19 Enrichment Process by Inorganic Acid

**Figure 1:**
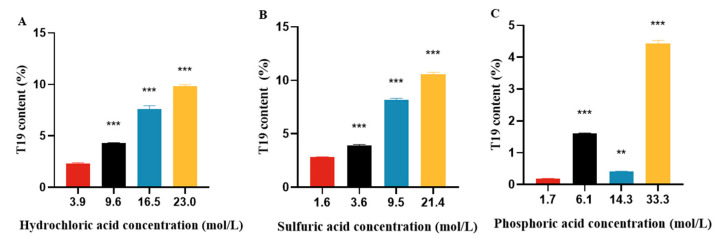
Comparison of determination results for three strong acids of (**A**) hydrochloric acid; (**B**) sulfuric acid; and (**C**) phosphoric acid. ** *p* < 0.01, *** *p* < 0.001, vs. the lowest acid concentration group.

We selected hydrochloric acid, sulfuric acid, and phosphoric acid to hydrolyze ginsenosides from ginseng stems and leaves using the method described in Section 3.2 at the same reaction time (1 h) and temperature (25 °C). From the experimental results, it can be seen that the effect of the phosphoric acid hydrolysis reaction is not good. The yield of T19 obtained from the hydrolysis reaction of 21.4 mol/L sulfuric acid and 23.0 mol/L hydrochloric acid is not significantly different. Considering the strong corrosiveness, oxidation, and danger of sulfuric acid, it is not easy to operate. Therefore, hydrochloric acid was chosen for the acid hydrolysis reaction to prepare T19. The results are shown in Figure 1.

#### 2.1.1. Effect of Different Hydrochloric Acid Molarity on the Hydrolysis Process

**Figure 2:**
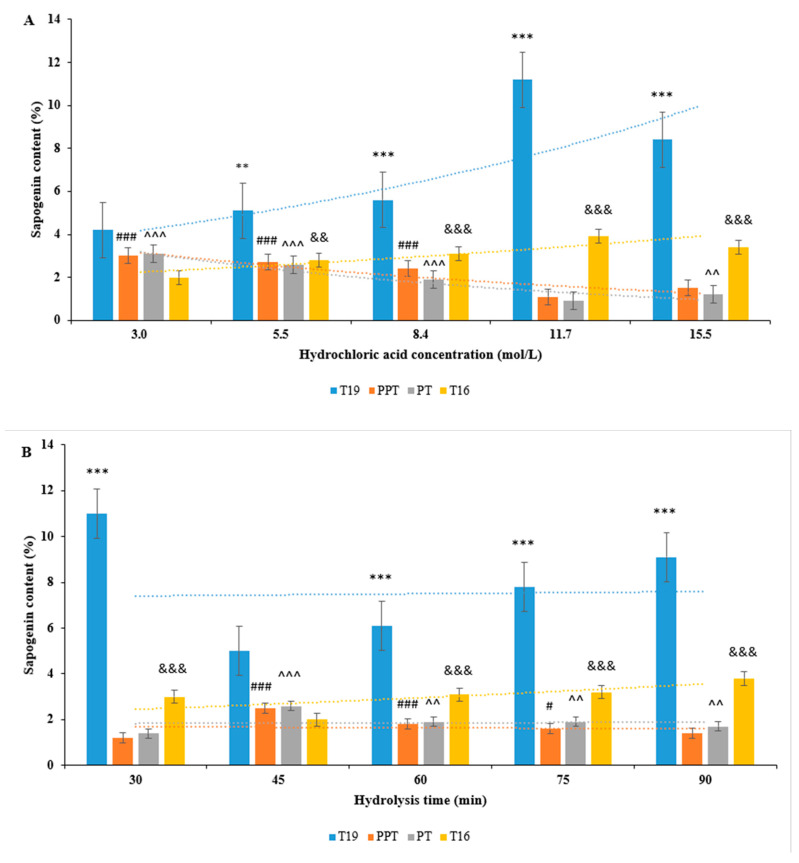

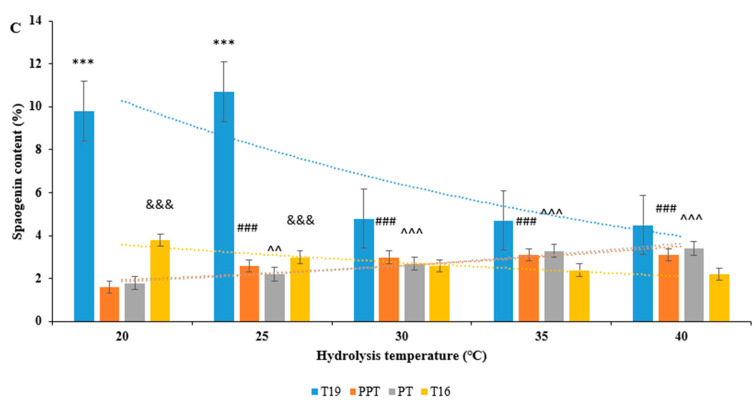
Comparison of hydrolysis results for ginseng sapogenins under different conditions of (**A**) hydrochloric acid mass fraction; (**B**) hydrolysis time; and (**C**) hydrolysis temperature. ** *p* < 0.01, *** *p* < 0.001 vs. the lowest T19 content; ^#^ *p* < 0.05, ^###^ *p* < 0.001 vs. the lowest PPT content; ^^^^ *p* < 0.01, ^^^^^ *p* < 0.001 vs. the lowest PT content; ^&&^ *p* < 0.01, ^&&&^ *p* < 0.001 vs. the lowest T16 content.

Under the conditions of hydrolysis temperature at 25 °C and hydrolysis time with 30 min, the content of T19, T16, PPT, and PT in the hydrolysis product was determined by the HPLC-ELSD method at the hydrochloric acid concentrations of 3.0, 5.5, 8.4, 11.7, and 15.5 mol/L, respectively. The results are shown in Figure 2A.

The results showed that with the increase in hydrochloric acid concentration, the T19 content significantly increased when the hydrochloric acid concentration was above 3.0 mol/L and reached its maximum at 11.7 mol/L. When the hydrochloric acid concentration approached 15.5 mol/L, carbonization may occur due to high acid concentration, which is not conducive to the conversion of T19. When the concentration of hydrochloric acid is less than 11.7 mol/L, the content of PPT and PT increases. At a concentration of 11.7 mol/L hydrochloric acid, the content of PT and PPT is the least. The content of them shows an opposite trend to that of T19, while the content of T16 shows the same trend as T19.

#### 2.1.2. Effect of Different Times on the Hydrolysis Process by Hydrochloric Acid

Under the conditions of hydrochloric acid concentrations of 11.7 mol/L and hydrolysis temperature with 25 °C, the content of T19, T16, PPT, and PT in the hydrolysis products was determined by the HPLC-ELSD method at hydrolysis times of 30, 45, 60, 75, and 90 min, respectively. The results are shown in Figure 2B.

The results showed that with the increase in hydrolysis time, the T19 content first decreased and then increased. The T19 content was highest at 30 min of hydrolysis reaction, decreased after 30 min, and increased again after 45 min. The content of T16 increases with the increase in T19, while the content of T19 decreases with the content of PT and PPT increasing. It is inferred that this may be due to the mutual transformation between T19 and PPT.

#### 2.1.3. Effect of Different Temperatures on the Hydrolysis Process by Hydrochloric Acid

Under the conditions of hydrochloric acid concentration of 11.7 mol/L and hydrolysis time of 30 min, the content of T19, T16, PPT, and PT in the hydrolysis products was determined by the HPLC-ELSD method at hydrolysis temperatures of 20, 25, 30, 35, and 40 °C, respectively. The results are shown in Figure 2C.

The results showed that the content of T19 was highest at 25 °C. As the ultrasound temperature increased, the content of T19 began to decrease. After the reaction temperature increased, the decreasing trend of T19 content tended to be gentle. As the reaction temperature increases, the content of PPT and PT shows an upward trend, which may be due to the higher temperature; it is more conducive to the desugar conversion of ginsenoside Rg1.

#### 2.1.4. Results of Orthogonal Test for Hydrochloric Acid Hydrolysis

We selected reaction temperatures, reaction times, and hydrochloric acid molarities as the main factors corresponding to four levels; following that, we conducted orthogonal experiments with a T19 content as the indicator. According to the results of the single-factor experiment, the selected concentration range of hydrochloric acid is 8.4–11.7 mol/L, the reaction time is 20–35 min, and the reaction temperature is 20–35 °C for the orthogonal experiment. The results are shown in Table 1.

By comparing the range Rj values of various influencing factors in the orthogonal experiment of ginsenoside hydrolysis of ginseng stems and leaves. By examining Table 1, experiment 15 yielded the best result (11.86 ± 0.22% of T19 content): hydrochloric acid concentration of 11.7 mol/L, hydrolysis time of 25 min, and a hydrolysis temperature of 30 °C. Considering this information and the data presented in Table 1, the optimized solution was A_4_B_2_C_3_.

Based on the investigation results, this hydrolysis process experiment was repeated three times with an RSD of less than 2%, indicating good repeatability of the experiment. The T19 content was about 12.16%.

### 2.2. Optimization of T19 Enrichment Process by Food Acids

#### 2.2.1. Effects of Different Food Acid Concentrations on the Hydrolysis Process

We selected commonly used food acids such as succinic acid, malic acid, tartaric acid, and citric acid and compared the effects of different acid types on hydrolysis using the method described in Section 3.2. Under the conditions of hydrolysis temperature at 80 °C and hydrolysis time with 2 h, the content of T19 in the hydrolysis product was determined by the HPLC-ELSD method.

**Figure 3:**
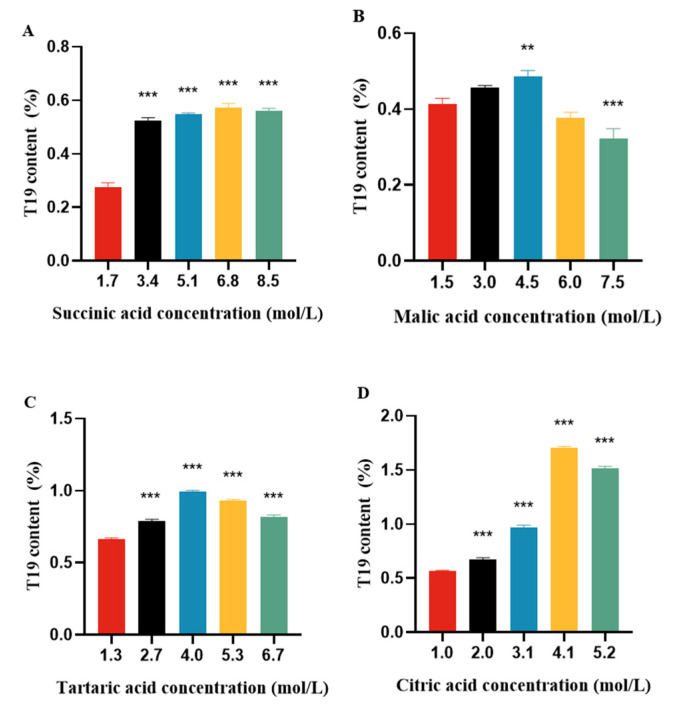
Comparison of determination results for (**A**) succinic acid molarity; (**B**) malic acid molarity; (**C**) tartaric acid molarity; and (**D**) citric acid molarity. ** *p* < 0.01, *** *p* < 0.001 vs. the lowest acid concentration group.

The results showed that citric acid had the best effect, and the content of T19 increased with the increase in citric acid concentration, far exceeding other food acids at 4.1 mol/L. The change in tartaric acid is significant but less than that of citric acid. Succinic acid and malic acid are similar, and there is not much difference in their ability to enrich T19 under different acidity conditions (Figure 3).

#### 2.2.2. Effect of Different Times on the Hydrolysis Process by Food Acid

Under the conditions of food acid concentration in 4.1 mol/L and hydrolysis temperature with 80 °C, the content of T19 in the hydrolysis products was determined by the HPLC-ELSD method at hydrolysis times of 30, 60, 90, and 120 min, respectively.

**Figure 4:**
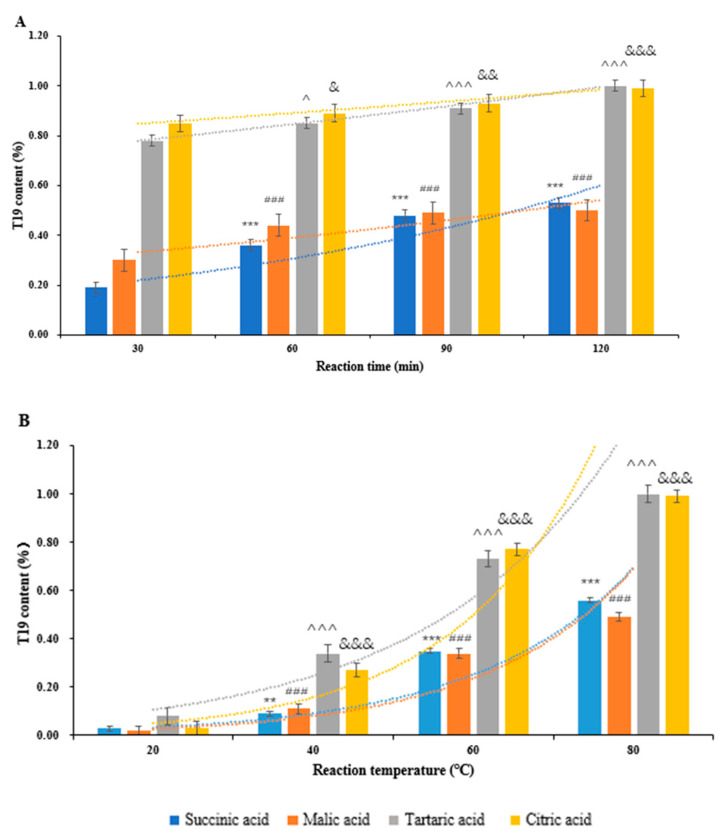
Comparison of determination results for four acids of (**A**) reaction time and (**B**) reaction temperature. ** *p* < 0.01, *** *p* < 0.001 vs. the lowest T19 content in succinic acid hydrolysis; ^###^ *p* < 0.001 vs. the lowest T19 content in malic acid hydrolysis; ^^^ *p* < 0.05, ^^^^^ *p* < 0.001 vs. the lowest T19 content in tartaric acid hydrolysis; ^&^ *p* < 0.05, ^&&^ *p* < 0.01, ^&&&^ *p* < 0.001 vs. the lowest T19 content in citric acid hydrolysis.

The results of different reaction times are shown in Figure 4A. By comparing different acids at different reaction times, it was found that the yield of citric acid-enriched T19 is similar to that of tartaric acid, and both tend to be similar at different reaction times. The succinic acid and malic acid, both of which have a significantly lower ability to enrich T19 than citric acid and tartaric acid, and the enrichment ability of succinic acid and malic acid in reaction time is relatively similar.

#### 2.2.3. Effect of Different Temperatures on the Hydrolysis Process by Food Acid

Under the conditions of food acid concentration of 4.1 mol/L and hydrolysis time of 2 h, the content of T19 in the hydrolysis products was determined by the HPLC-ELSD method at hydrolysis temperatures of 20, 40, 60, and 80 °C, respectively.

The results of T19 enrichment about four types of food acids at different temperatures are shown in Figure 4B. It was found that the yield of all food acids was extremely low at low temperatures, and as the temperature increased, the yield of all acid-enriched T19 increased significantly. Among them, the curves of citric acid and tartaric acid are relatively close, with similar high enrichment values at 80 °C.

#### 2.2.4. Results of Orthogonal Test for Citric Acid Hydrolysis

Combined with the single factor conditions for T19 enrichment, the factors were selected: citric acid concentration (3.6–5.2 mol/L), reaction time (60–180 min), and reaction temperature (70–100 °C). According to the principle of orthogonal experiment design, the orthogonal analysis of three factors and three levels was adopted on the basis of single factor tests.

As can be seen from Table 2, R_A_ > R_C_ > R_B_, indicating that the relationship between the influencing factors is:

A (citric acid) > C (temperature) > B (time).

The significance levels of the three factors, without considering their interaction, were A_3_B_2_C_3_; that is, the concentration of citric acid was 5.2 mol/L, the reaction time was 120 min, and the reaction temperature was 100 °C.

Based on the investigation results, this hydrolysis process experiment was repeated three times with an RSD of less than 2%, indicating good repeatability of the experiment. The T19 content was about 1.9%.

### 2.3. Molecular Docking Test of T19 to Its Binding Targets

**Figure 5:**
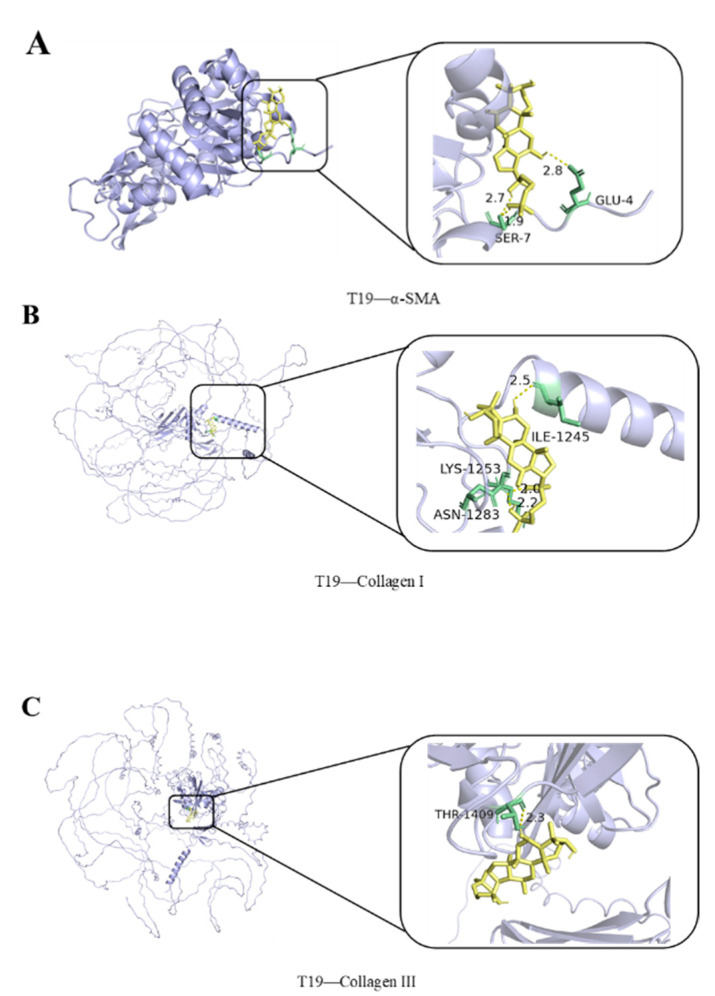
Molecular docking showed the direct binding analysis of T19 to its potential target proteins (**A**) alpha-smooth muscle actin (α-SMA, UniProt ID: P62736); (**B**) Collagen alpha-1 (I) chain (Collagen I, UniProt ID: P02452); and (**C**) Collagen alpha-1 (III) chain (Collagen III, UniProt ID: P02461).

For further exploring the possible mechanism T19 functions through, a molecular docking test was performed to assess the binding potentiality of T19 to its target. The 3D structure data of alpha-smooth muscle actin (α-SMA, UniProt ID: P62736), Collagen alpha-1 (I) chain (Collagen I, UniProt ID: P02452), and Collagen alpha-1 (III) chain (Collagen III, UniProt ID: P02461) was downloaded from the Uniport database. The binding affinities between T19 and the core targets above were tested by AutoDock Vina. After the analysis, hydrogen bonds would form between T19 and amino acid residues of SER-7 (1.9 and 2.7 Å), as well as GLU-4 (2.8 Å) of α-SMA (Figure 5A). The binding affinity of T19 and α-SMA was −7.0 kcal/mol (Figure 5B). As for Collagen I, the hydrogen bonds formed with amino acid residues ILE-1245 (2.5 Å), LYS-1253 (2.0 Å), and ASN-1283 (2.2 Å), making the binding affinity of −7.8 kcal/mol (Figure 5B). At last, the binding was also determined between T19 and Collagen III with the amino acid residues of THR-1409 (2.3 Å) and a binding affinity of −6.0 kcal/mol (Figure 5C). Taken together, our data suggested that T19 potentially binds to its target proteins directly.

### 2.4. Establishment of H9c2 Cardiomyocyte Injury Model

**Figure 6:**
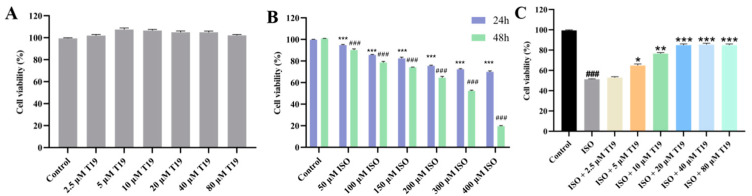
H9c2 cardiomyocyte survival rate measured by MTT assay: (**A**) effect of T19 on H9c2 cell survival rate; (**B**) effect of ISO on H9c2 cell survival rate at 24 h and 48 h (*n* = 3, mean ± SD; ^###^ *p* < 0.001; * compared with the 24 h group, *** *p* < 0.001); and (**C**) effect of T19 on ISO-induced H9c2 cell survival rate (*n* = 3, mean ± SD; ^###^ *p* < 0.001; * compared with the model group, * *p* < 0.05, ** *p* < 0.01, *** *p* < 0.001).

As shown in Figure 6A, T19 obtained by citric acid concentrations of 2.5–80 μM had no significant toxic effects on cells compared with the blank group. However, when the ISO concentration was 300 μM for 48 h treatment, the cell growth was significantly inhibited in the model group compared with the blank control group, and the cell survival rate decreased to 50.5%, indicating that myocardial cells were damaged (Figure 6B). Therefore, to establish a myocardial cell injury model, the H9c2 cells were treated with 300 µM ISO for 48 h. Figure 6C illustrates the protective effect of T19 at concentrations of 2.5–80 μM on ISO-induced cardiomyocyte injury. Compared with the ISO group, the protective effect of T19 on cardiomyocytes increased in a dose-dependent manner, and the protective effect was most significant at a T19 concentration of 20 µM.

### 2.5. Effect of T19 on the Activity of LDH and CK-MB Enzymes in ISO-Induced H9c2 Cardiomyocytes

**Figure 7:**
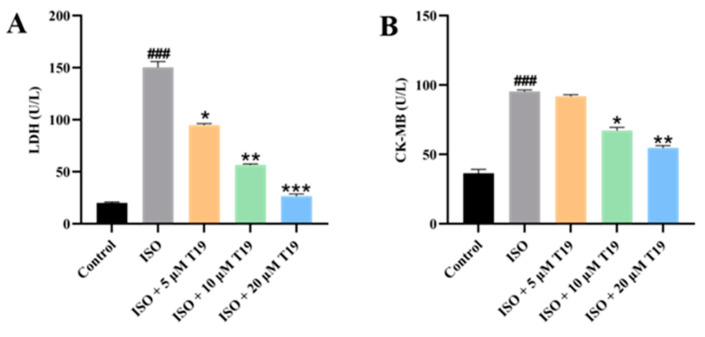
Effect of T19 on the activity of LDH and CK-MB enzymes in ISO-induced H9c2 cardiomyocytes: (**A**) LDH and (**B**) CK-MB (*n* = 3, mean ± SD; ^###^ *p* < 0.001; * compared with the model group, * *p* < 0.05, ** *p* < 0.01, *** *p* < 0.001).

Compared with the blank control group, the levels of LDH and CK-MB in the model group were significantly increased. However, the levels of LDH and CK-MB in the low-dose, medium-dose, and high-dose experimental groups were significantly reduced compared with the model group, as shown in Figure 7.

### 2.6. Effects of T19 on Oxidative Stress Injury in ISO-Induced H9c2 Cardiomyocytes

**Figure 8:**
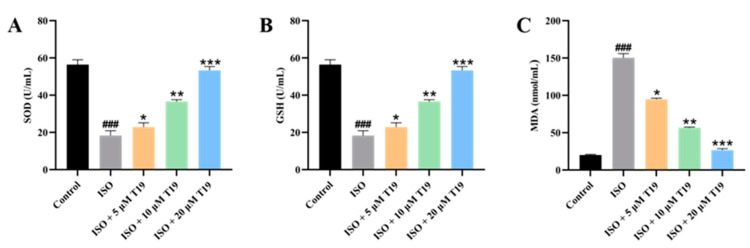
Effect of T19 on oxidative stress levels in ISO-induced H9c2 cardiomyocytes: (**A**) SOD; (**B**) GSH; and (**C**) MDA (*n* = 3, mean ± SD, ^###^ *p* < 0.001; * compared with the model group, * *p* < 0.05, ** *p* < 0.01, *** *p* < 0.001).

Compared with the blank control group, the activity of SOD and the content of GSH in the model group were significantly decreased, and the content of MDA was significantly increased. However, compared with the model group, the activity of SOD and the content of GSH were significantly increased in the low-dose, medium-dose, and high-dose experimental groups, while MDA content was significantly decreased, as shown in Figure 8.

### 2.7. Protein Expression of Collagen I, Collagen III, and α-SMA Was Detected by Western Blot

**Figure 9:**
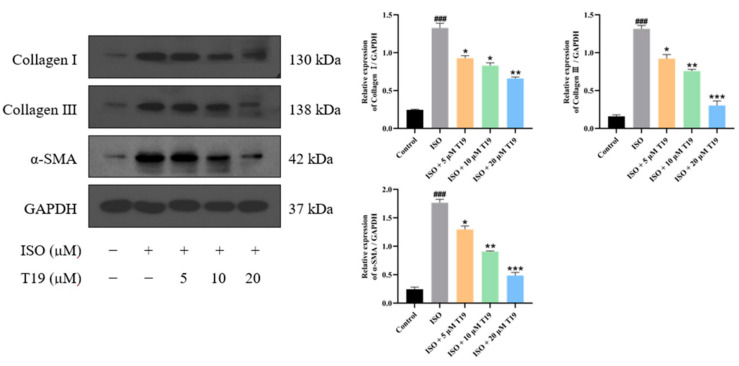
Protein expression of fibrosis markers in ISO-induced cardiomyocytes by T19 (*n* = 3, mean ± SD, ^###^ *p* < 0.001; * compared with the model group, * *p* < 0.05, ** *p* < 0.01, *** *p* < 0.001).

As shown in Figure 9, the protein expressions of Collagen I, Collagen III, and *α*-SMA in the model group were significantly increased compared with the control group. However, compared with the model group, T19 reduced the protein expression of Collagen I, Collagen III, and *α*-SMA in H9c2 cells in a dose-dependent manner.

### 2.8. mRNA Expression of Collagen I, Collagen III, and α-SMA in Cardiomyocytes Detected by PCR

**Figure 10:**
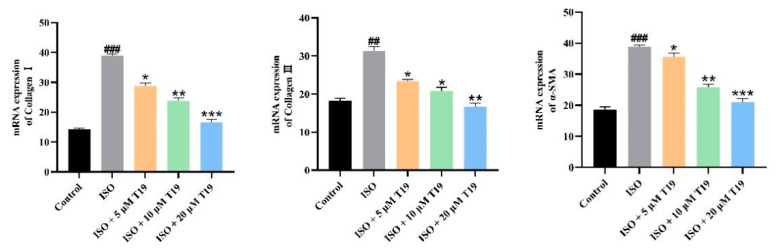
mRNA expression of fibrosis markers in ISO-induced cardiomyocytes by T19 (*n* = 3, mean ± SD, ^##^ *p* < 0.01, ^###^ *p* < 0.001; * compared with the model group, * *p* < 0.05, ** *p* < 0.01, *** *p* < 0.001).

As shown in Figure 10, mRNA expressions of Collagen I, Collagen III, and *α*-SMA in the model group were significantly increased compared with the blank control group. However, compared with the model group, T19 reduced the mRNA expression of Collagen I, Collagen III, and *α*-SMA in H9c2 cells in a dose-dependent manner.

## 3. Materials and Methods

### 3.1. Chemicals and Materials

Ginseng stem and leaf saponin was obtained from Liaoning Fushun Xintai Ginseng Health Care Product Co., Ltd. (Fushun, China). Methanol (chromatographic grade) and other solvents (analytical grade) were purchased from Kangkede (Tianjin, China). Water for HPLC analysis and sample preparation was obtained from Wahaha Group Co., Ltd. (Hangzhou, China).

### 3.2. Preparation of Acid Hydrolysis Samples

Preparing a 100 mg/mL solution of ginseng stem and leaf saponins. The ginseng stem and leaf saponins solid powder were dissolved in various acidic solutions to obtain acid ginsenosides. The effects of acid type, concentration, reaction time, and reaction temperature on the content of T19 in acid hydrolysate were investigated. After the reaction was completed, aqueous sodium hydroxide was added to adjust back to neutrality. After centrifugation at 4000 rpm for 5 min, the precipitate was washed 3 times with iced water and was dried to constant weight in an oven at 70 °C. The content of T19 in the hydrolysis products was determined by the HPLC-ELSD method.

### 3.3. Orthogonal Experimental Design

To further explore the T19 preparation with hydrochloric acid and citric acid hydrolysis, the process was optimized. Three factors were selected in the orthogonal experiment, including acid concentration, reaction temperature, and reaction time. Based on the single-factor level experiments, to explore the most suitable preparation conditions. The factor levels are shown in Table 3 and Table 4.

### 3.4. Instrumentation and Chromatographic System

**Figure 11:**
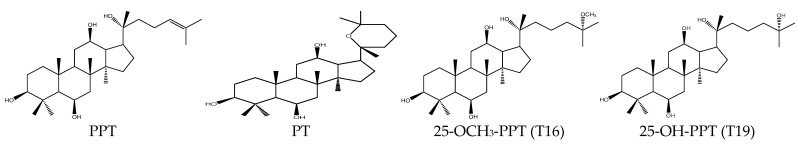
Chemical structures of four ginseng sapogenins.

Analyses were performed on an HPLC system (Knauer, Berlin, Germany) equipped with a Kromasil-C18 chromatography column (4.6 mm × 250 mm, 5 μm, Akzo Nobel, Arlöv, Sweden) and an evaporative light-scattering detector (ELSD, Shanghai Tongwei Analysis Technology Co., Ltd., Shanghai, China). The probe temperature of ELSD was set to 50 ℃, and the nebulizer for nitrogen gas was 3 L/min. The flow rate was 1.0 mL/min, and the injection volume was 20 μL. The column temperature was 25 ℃. The mobile phase consisted of (A) water and (B) methanol. The elution procedure of the analytical method is 0~30 min, 70% B; 30~63 min, 80% B, based on the previous methodological development by our research group [26]. The method can simultaneously determine the content of 25-OH-PPT (T19), 25-OCH_3_-PPT (T16), PPT, and PT. The structures of the four substances were shown in Figure 11.

### 3.5. Molecular Docking Assay

Molecular docking analysis was performed to predict the possible binding site of T19 on the target. The structural data of the target proteins was downloaded from the Universal Protein (Uniport) database. The chemical structure data of T19 were drawn by Chemdraw 20.0 and Chem3D 22.0. The PDB file obtained was firstly converted to a pdbqt file for the next process. AutoDock Tools 1.5.7 were then used to apply the molecular docking analysis between diosgenin and target proteins. The results of docking were visualized and displayed by PyMOL 2.5.7.

### 3.6. Cell Culture and Anti-Myocardial Cell Injury Activity Assay

#### 3.6.1. Cell Culture

The H9c2 cardiomyocytes, purchased from Meilun Biotechnology Company (Suzhou, China), were cultured in Dulbecco’s modified Eagle’s medium (DMEM, Gibco, New York, NY, USA) supplemented with 10% fetal bovine serum (FBS, Gibco, NY, USA) and 1% penicillin–streptomycin (P/S, Genview, Changsha, China) in a 37 ℃ incubator with a 5% carbon dioxide (CO_2_) atmosphere. Cells were divided into control, isoproterenol (ISO), and administration groups. The ISO group received treatment with ISO (300 μM) for 48 h, except for the control group. In the administration group, cells were pretreated with the corresponding concentration of T19 (2.55, 10, 20, 40, and 80 μM) for 4 h before ISO treatment.

#### 3.6.2. Cell Viability Assay

ISO can stimulate myocardial cells, leading to myocardial hypertrophy, myocardial cell damage, and myocardial fibrosis. Hence, the protective effect of T19 on ISO-induced H9c2 cardiomyocyte injury was studied. The effects of T19 on H9c2 cardiomyocytes cell viability were investigated using the MTT assay.

Cardiomyocytes were incubated in a 96-well plate (5 × 10^4^ cells/well) and plated for 24 h. T19 was dissolved in cell-grade DMSO and prepared as a mother liquor with a final concentration of 1 × 10^4^ µM in advance. After the cells were completely attached to the wall, T19 was diluted at gradients of 2.5, 5, 10, 20, 40, and 80 μM, and the cell viability was detected for 24 h. Following treatment, each well was supplemented with 10 μL of MTT solution and incubated at 37 °C for an additional 4 h and then mixed with DMSO sequentially. Absorbance readings were taken at a wavelength of 490 nm using a microplate reader. The experiment was repeated three times, and the cell survival rate of each experimental group was calculated by the following formula:

Cell survival rate = OD _experimental group_/OD _blank group_ × 100%

Different concentrations (50, 100, 150, 200, 300, and 400 µM) of ISO were used to induce H9c2 for 24 and 48 h to construct myocardial injury models. In order to explore the effect of T19 on ISO-induced cardiotoxicity of H9c2 cells, T19 (2.5–80 µM) protection was performed for 4 h, and then ISO (300 µM) treatment and T19 coincubation for 48 h were performed. Subsequently, MTT assays were performed as previously described.

### 3.7. Determination of LDH, CK-MB, SOD, GSH, and MDA Content

The supernatant of H9c2 cardiomyocytes after treatment was collected, and assays for lactate dehydrogenase (LDH), creatine kinase isoenzyme MB (CK-MB), superoxide dismutase (SOD), glutathione (GSH), and malondialdehyde (MDA) were measured according to instructions of the Nanjing Jiancheng kit (Nanjing, China).

### 3.8. Western Blot (WB) Analysis

After the treatment, RIPA buffer was used to extract the total protein. The protein concentration of each group was measured by the BCA protein quantitative kit. SDS loading buffer protein was added to quantify the protein concentrations of all samples and transferred onto a nitrocellulose filter (NC) membrane. The membrane was blocked in 5% milk for 1.5 h and then incubated overnight with antibodies against Collagen I, Collagen III, α-SMA, and GAPDH at various dilutions. GAPDH is used as a loading control antibody. After washing with TBST, the membrane was then incubated with secondary antibodies (goat anti-rabbit) for 1 h. 

### 3.9. Real-Time Quantitative RT-PCR

The Servicebio^®^RT First Strand cDNA Synthesis Kit (Servicebio, Wuhan, China) was used to reverse transcribe RNA into cDNA. Their action was detected by a 2 × SYBR Green qPCR Master Mix (None ROX) before performing RT-PCR with a LightCycle^®^ 480 II fluorescence quantitative PCR instrument (Roche, Basel, Switzerland). Normalizing against the reference gene β-actin (mRNA), the relative quantitative 2^−∆∆Ct^ method was implemented to evaluate relative gene expression. The *t*-test was used to assess differences in expression. The primer sequences were designed using Primer 5.0.

PCR primer sequences were as follows:

*α*-SMA forward primer ACCATCGGGAATGAACGCTT and reverse primer CTGTCAGCAATGCCTGGGTA;

Collagen I forward primer CCCAGCGGTGGTTATGACTT and reverse primer TCGATCCAGTACTCTCCGCT;

Collagen III forward primer AATATGTCCACAGCCTTCTACACC and reverse primer ACCCATTCCTCCGACTCCA;

GAPDH forward primer CTGGAGAAACCTGCCAAGTATG and reverse primer GGTGGAAGAATGGGAGTTGCT.

### 3.10. Statistical Analysis

The experiment was replicated three times, experimental data were analyzed using GraphPad Prism 9.0, and results were represented as the mean and standard deviation (SD). The data were evaluated by one-way analysis of variance (ANOVA) followed by a Duncan’s test, and the difference was considered significant when *p* < 0.05.

## 4. Discussion

In this study, in order to optimize the T19 enrichment process, we conducted a hydrolysis process study on ginseng stems and leaves ginsenosides, optimizing acid types, acid concentrations, ultrasound time, and ultrasound temperature. The hydrolysis results showed that with the change in acid concentration, hydrolysis temperature, and hydrolysis time, the T19 content showed an opposite trend to that of PT and PPT, which was speculated to be due to the mutual transformation between T19 and PPT, while the T16 content showed the same trend as that of T19. Meanwhile, research has found that using hydrochloric acid to hydrolyze ginsenosides in ginseng stems and leaves can increase the content of T19 to 12.16%. When using edible citric acid, the maximum content of T19 is 1.9%. It can be seen that the strength of acidity has a significant impact on the content of T19 produced. However, using citric acid for hydrolysis has higher safety and non-toxic properties. Compared with the previous enrichment of T19 by acid hydrolysis, this study did not involve methanol, ethyl acetate, and other organic solvents, which provides a new idea for the future development of T19 in the direction of functional food.

The protective effect of T19 on H9c2 cardiomyocytes was evaluated with the ISO-induced H9c2 cardiomyocyte injury model.

ISO-induced cardiomyocyte injury is an experimental model widely used in the screening of anti-myocardial fibrosis drugs. ISO injury can cause cell membrane rupture and leakage of intracellular substances, including cytoplasmic enzymes LDH and CK-MB in various tissues. There is a significant positive correlation between elevated levels of LDH and CK-MB enzymes and myocardial injury. Therefore, LDH and CK-MB can be used as biomarkers to evaluate myocardial cell injury [27,28]. In this study, the levels of LDH and CK-MB were significantly increased, and cell survival was reduced in the ISO group, indicating damage to cardiomyocytes. However, compared with the ISO group, T19 pretreatment significantly reduced the levels of LDH and CK-MB and increased cell survival rate. Hence, T19 pretreatment significantly reduced this damage and protected cardiomyocytes from ISO-induced myocardial injury.

A large number of studies have shown that ISO-induced cardiomyocyte injury can lead to increased levels of oxidative stress in cells and promote the production of a large number of oxygen free radicals, thus affecting the normal physiological function of cardiomyocytes [29,30]. It is inferred that the activity of T19 is related to the antioxidant activity. The key antioxidant enzyme SOD and non-enzymatic antioxidant GSH are those that neutralize superoxide radicals and decompose hydrogen peroxide into harmless molecules [31]. Therefore, SOD and GSH are considered effective markers of oxidative damage [31]. In this study, T19 pretreatment effectively restored the activity of antioxidant enzymes SOD and GSH in cardiomyocytes. MDA is a lipid peroxide metabolite and a marker of cell damage caused by oxygen free radicals [32]. MDA levels were reduced in a dose-dependent manner in the T19 pretreatment group. It was also shown that T19 can protect against ISO-induced cardiomyocyte injury by reducing the levels of oxidative stress. Together, the findings suggest that T19 can effectively reverse the loss of antioxidative activity and can protect against ISO-induced cardiomyocyte injury by reducing oxygen free radicals and levels of oxidative stress.

ISO-induced cardiomyocyte injury is not only manifested as elevated oxidative stress but also leads to myocardial fibrosis [33]. Myocardial fibrosis is a pathological marker of many cardiovascular diseases, mainly due to the large amount of collagen fiber deposition in the extracellular matrix (ECM). Collagens I and III are the main components of cardiac ECM. *α*-SMA is pathologically manifested as fibrosis and differentiates fibroblasts into myofibroblasts. This triggers ECM signals and accelerates heart damage [34]. Meanwhile, the molecular docking results show that the binding affinity of T19 and α-SMA was −7.0 kcal/mol (≤7.0 kcal/mol), which indicates that T19 and α-SMA have good binding ability; as for Collagen I, the binding affinity was −7.8 kcal/mol (≤7.0 kcal/mol), indicating a strong binding activity of T19 and Collagen I; the binding energy of T19 and Collagen III was −6.0 kcal/mol (≤5.0 kcal/mol), indicating that T19 has a good combination with Collagen III. Therefore, Collagen I and III and *α*-SMA are considered biomarkers of myocardial fibrosis [35]. To confirm that T19 pretreatment can improve ISO-induced myocardial fibrosis, this study first used Western blot experiments and PCR technology to demonstrate that T19 reduces the protein expression of these fibrosis markers. In conclusion, T19 pretreatment can effectively improve the oxidative stress response and fibrosis in H9c2 cardiomyocytes, thereby alleviating ISO-induced cardiomyocyte injury.

## 5. Conclusions

In this study, T19 was enriched from ginseng stems and leaves ginsenosides with the acid hydrolysis method, and the protective effect of T19 on ISO-induced H9c2 cardiomyocyte injury was studied. It is speculated that the protective mechanism of T19 may be the regulation of LDH and CK-MB enzyme activity and the improvement of oxidative stress levels. These findings provide new perspectives to study the pharmacological mechanisms of T19. It was helpful for the isolation of anti-myocardial fibrosis compounds in ginseng stems and leaves.

## Figures and Tables

**Table 1:** The L9(3^4^) analysis of orthogonal experiment results.

Number	A (Hydrochloric Acid Concentration/mol/L)	B (Hydrolysis Temperature/℃)	C (Hydrolysis Time/min)	T19 Content/%
1	8.4	20	20	3.49 ± 0.16
2	8.4	25	25	5.67 ± 0.23
3	8.4	30	30	9.47 ± 0.32
4	8.4	35	35	9.86 ± 0.25
5	9.5	20	25	4.81 ± 0.14
6	9.5	25	30	8.14 ± 0.23
7	9.5	30	35	9.81 ± 0.20
8	9.5	35	20	9.19 ± 0.19
9	10.6	20	30	1.43 ± 0.08
10	10.6	25	35	3.64 ± 0.11
11	10.6	30	20	7.90 ± 0.20
12	10.6	35	25	5.87 ± 0.12
13	11.7	20	35	10.05 ± 0.35
14	11.7	25	20	10.10 ± 0.44
15	11.7	30	25	11.86 ± 0.22
16	11.7	35	30	9.62 ± 0.34
k_1_	7.12	4.95	7.67	
k_2_	7.99	6.89	7.05	
k_3_	4.71	9.76	7.17	
k_4_	10.41	8.64	8.34	
R	5.70	4.81	1.29	

**Table 2:** The L9(3^3^) analysis of orthogonal experiment results.

Number	A (Citric Acid Molarity/mol/L)	B (Hydrolysis Time/min)	C (Hydrolysis Temperature/℃)	T19 Content/%
1	3.6	60	70	0.73 ± 0.03
2	3.6	120	85	1.14 ± 0.04
3	3.6	180	100	0.91 ± 0.02
4	4.4	120	70	1.19 ± 0.04
5	4.4	180	85	1.65 ± 0.07
6	4.4	60	100	1.67 ± 0.05
7	5.2	180	70	1.62 ± 0.04
8	5.2	60	85	1.55 ± 0.03
9	5.2	120	100	1.91 ± 0.08
k_1_	0.93	1.32	1.18	
k_2_	1.50	1.41	1.45	
k_3_	1.69	1.39	1.50	
R	0.76	0.09	0.32	

**Table 3:** The L9(3^4^) orthogonal factor horizontal design in hydrochloric acid.

Level	A (Hydrochloric Acid Molarity/mol/L)	B (Reaction Time/min)	C (Reaction Temperature/℃)
1	8.4	20	20
2	9.5	25	25
3	10.6	30	30
4	11.7	35	35

**Table 4:** The L9(3^3^) orthogonal factor horizontal design in citric acid.

Level	A (Citric Acid Molarity/ mol/L)	B (Reaction Time/min)	C (Reaction Temperature/℃)
1	3.6	60	70
2	4.4	120	85
3	5.2	180	100

## Data Availability

The data that support the findings of this study are available from the corresponding author upon reasonable request.
